# Supplementary material for: Dermal Fibroblasts Internalize Phosphatidylserine-Exposed Secretory Melanosome Clusters and Apoptotic Melanocytes
Source: Int J Mol Sci. 2020 Aug 12;21(16):5789. doi: 10.3390/ijms21165789 (PMC7461560; doi:10.3390/ijms21165789)
Supplement: Supplementary file 1 [file ijms-21-05789-s001.zip › ijms-833817 supplementary/Supplementary Materials - ijms-833817.docx]

**SUPPLEMENTARY MATERIALS**


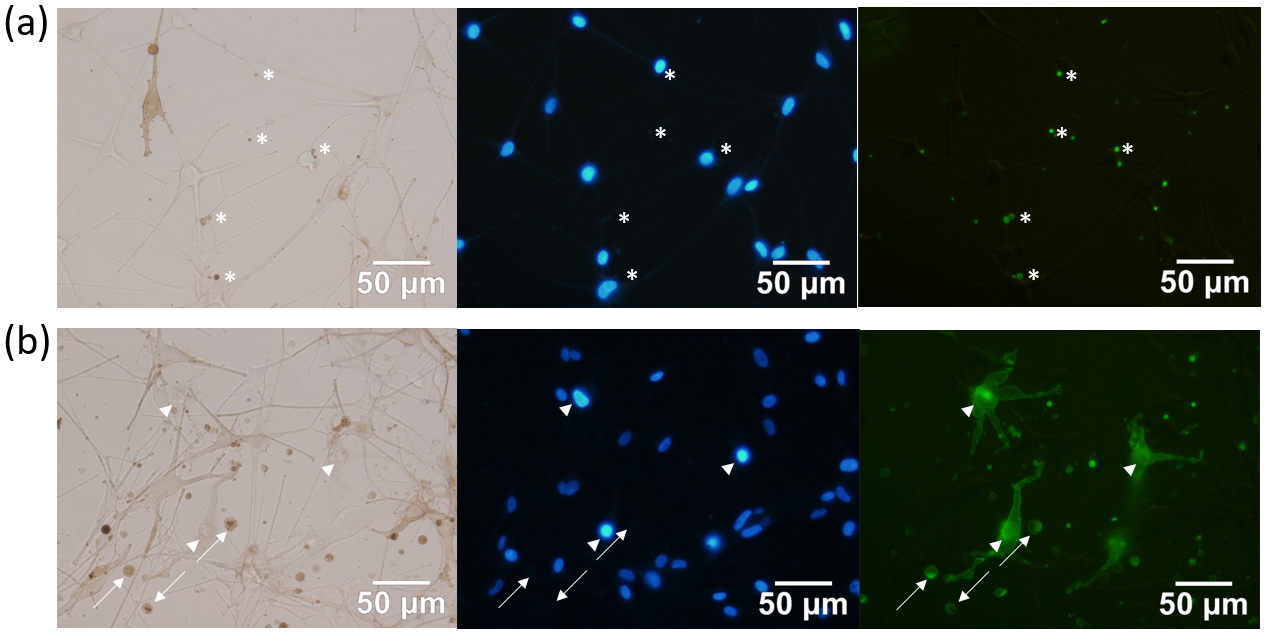


**Figure S1.** The same fields of (a) non-apoptotic melanocytes and (b) actinomycin D-induced apoptotic melanocytes (24 h incubation at 4 μg/ml) are shown with (left panels) bright field, (middle panels) DNA staining with Hoechst 33342 and (right panels) immunofluorescence staining of PtdSer by Annexin V. Asterisks in (a) indicate secretory melanosome clusters. Arrows in (b) indicate apoptotic bodies without nuclear DNA. Arrow-heads in (b) indicate apoptotic melanocytes with shrunken nuclei and PtdSer exposure on the cell membrane.


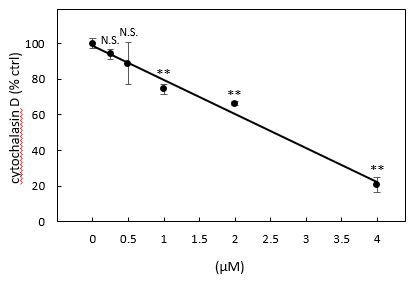


**Figure S2.** Cell viability determined using the MTS assay after treatment with or without various concentrations of cytochalasin D for 24 h. Data represent means ± SD; ** = p<0.01 versus 0 μM; N.S. = Not significant.

**Figure V1 (Video)** Time-lapse imaging at 9,000-fold speed showing fibroblasts actively moving towards and engulfing the secreted melanosome clusters.
